# Design and development of a cost-effective buoy using 3D printing for coastal monitoring

**DOI:** 10.1016/j.ohx.2025.e00657

**Published:** 2025-05-22

**Authors:** Simón F. Nogueira, Alejandro J. Vitale, Sibila A. Genchi, Agustina Roth, Steven Martínez Vargas, Agustin Siben, Lucas Nuciari, Gerardo M.E. Perillo

**Affiliations:** a Departamento de Ingeniería, Universidad Nacional del Sur (UNS), Bahía Blanca, Argentina; b Instituto Argentino de Oceanografía (IADO), CONICET-UNS, B8000FWB, Bahía Blanca, Argentina; c Departamento de Ingeniería Eléctrica y de Computadoras, UNS, Bahía Blanca, Argentina; d Departamento de Geografía y Turismo, UNS, Bahía Blanca, Argentina; e Departamento de Geología, UNS, Bahía Blanca, Argentina

**Keywords:** Monitoring buoy, Continental/coastal waters, 3D printing, Low-cost, Open-source

## Abstract

An adequate knowledge of water (and air) environment parameters in ocean and coastal areas becomes of crucial importance for management practices. The behavior of oceans and coasts covers a large range of spatial and time scales that leads to the importance of high-frequency and widespread monitoring by using cost-effective platforms. This article proposes the design, development and deployment of a low-cost, small and compact buoy platform for coastal monitoring, which was built almost entirely on 3D printing technology in a modular way. The buoy called EMAC buoy -V3.0- (EMAC: Estación de Monitoreo Ambiental Costero) was significantly improved in the last few years based on cost-effective optimization and physical constraints inherent to coastal waters, providing the advantages of simplicity and flexibility. This study considered oceanographic and meteorological measurements of a buoy moored in the San Matías Gulf on the northern Patagonian Continental Shelf.

## Highlights

• A low-cost, modular buoy built with 3D printing for coastal monitoring is proposed.
• 3D printing reduces costs while maintaining operational viability and flexibility.
• A repository of mechanical and electronic designs and source codes is presented.

## Graphical abstract

**Figure:**
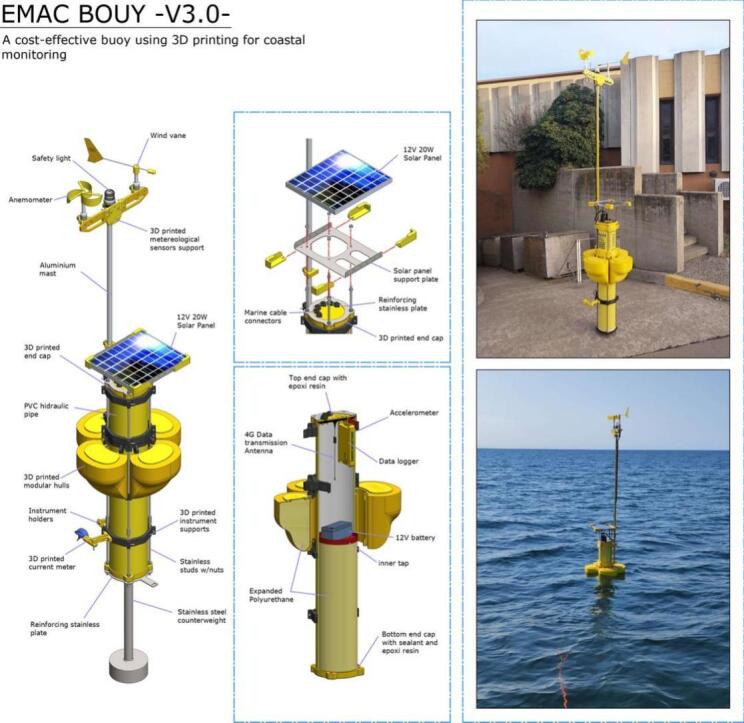

**Table:** 

Hardware name	EMAC buoy -V3.0-
Subject area	Engineering and materials science Environmental, planetary and agricultural sciences
Hardware type	Field measurement and sensors
Closes commercial analog	Tideland SB138-P Sentinel Buoy (Not similar in size)
Opel Licence Source	GNU GPL V3.0
Cost of hardware	USD 4392 (Assembling and 3D printing are not included in the cost)
Source File Repository	https://data.mendeley.com/datasets/84fygz2txp

## 1 Hardware in context

The ocean, which covers 71 % of the Earth's surface, is the most important life-supporting system [1]. At the same time, the ocean is a key determinant of Earth's climate system [2,3]. Ocean and humans are historically closely connected [4] since the former provides benefits such as food, hydropower generation, recreational tourism, etc. About 40 % of the total population lives within 100 km of the coast [5] with the consequences that this would entail. A clear example of the need for changing human management toward the ocean and coastal seas was proposed by the United Nations which launched the UN Decade of Ocean Science for Sustainable Development (2021–2030) [6]. Therefore, an adequate knowledge of water (and air) environment parameters in ocean and coastal areas becomes of crucial importance for management practices.

The behavior of oceans and coasts covers a large range of spatial and time scales [7]. This condition leads to the importance of high-frequency and widespread monitoring by using cost-effective platforms. The access to low-cost instrumentation remains a limiting factor in coastal ocean monitoring [8]. The commercial buoy platforms are still expensive (hundreds of thousands of dollars, depending on the on-board sensors) [9] for research purposes, even more in developing countries. There is increasing interest in research developments based on the idea of collecting data using low-cost buoy systems [10]. For instance, Schmidt et al. (2018) [11] proposed the design, construction and deployment of a low-cost (<£ 5000) monitoring buoy for coastal aquaculture farms. Laun and Pittman (2019) [12] focused on designing, fabricating and deploying a small, free-floating, networked buoy for persistent ocean monitoring, for less than $500 USD. More recently, Lu et al. (2022) [13] designed and implemented a low-cost and easy-to-build buoy system using artificial intelligence that autonomously measures the related water quality data for an offshore aquaculture environment.

3D Printing (3DP) is emerging as an enabling technology and its low-cost and increased capabilities are given unprecedented opportunities for observational oceanography [14]. This technology is being used in several ocean/coastal applications such as autonomous underwater or surface vehicles, drifters, and replicas of marine organisms and coral reefs, but its potential for buoy platforms is still poorly explored. For example, Agade and Bean (2023) [15] presented a free-flowing buoy for monitoring flowing stream environments mostly built with 3DP (i.e., housing and circuit boards). Kodaira et al. (2023) [16] focused on a wave buoy design for wave-ice interactions applying 3DP as rapid prototyping technology.

This article proposes the design, development and deployment of a low-cost, small and compact buoy platform for coastal monitoring, which was built almost entirely on 3DP technology in a modular way. The buoy called EMAC buoy -V3.0- (EMAC: Estación de Monitoreo Ambiental Costero [17]) was significantly improved in the last few years based on cost-effective optimization and physical constraints inherent to coastal waters. The earlier versions were originally designed to monitor lakes and estuaries and their description can be found in detail in Vitale et al. (2018) [10]. Currently, the EMAC buoys -V3.0- are deployed in different sites of the Argentine Sea [17]. For the purposes of this study, oceanographic and meteorological measurements of a buoy moored in the San Matías Gulf on the northern Patagonian Continental Shelf were considered.

## 2 Hardware description

As mentioned above, the EMAC buoy -V3.0- is the result of improvements, prioritizing physical and kinematic constraints in response to the complex coastal waters, with high-reliability and low-cost. Most of the mechanical and electronic components of the buoy were designed and developed by a team of researchers from the Instituto Argentino de Oceanografía (IADO-CONICET, Argentina). 3DP was used to create several parts of the buoy. In addition, readily available components were bought and merged into the design. The key hardware designs will be described in detail below.

### 2.1 Architecture and design of the buoy

**Fig. 1:**
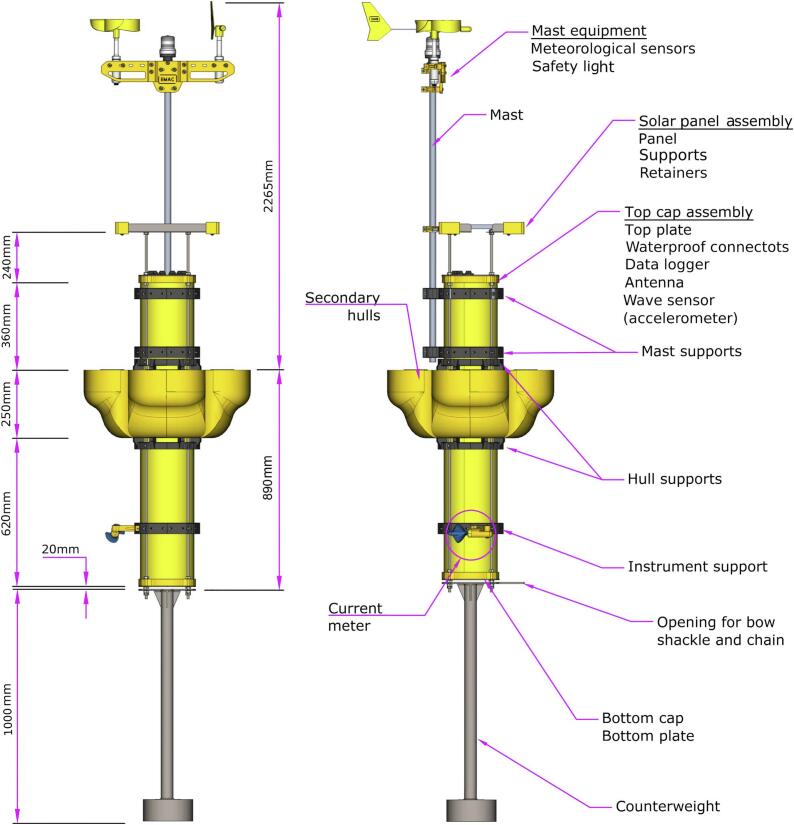
EMAC buoy -V3.0- schematic and its constitutive parts and dimensions.

Table 1 describes the technical specifications of the EMACvv h buoy -V3.0-. The main hull consists of a machined PVC pipe of 200 mm in diameter and 1230 mm in height, which is partially filled with expanded polyurethane and closed with a pair of 3D printed ABS end caps (Fig. 1). There are six waterproof through-hole connectors (and four pins) on the top end cap for electrical purposes. The pieces are assembled with the help of four long threaded studs with double stainless steel nuts (Fig. 1). Inside the main hull there are major components including data logger, battery, data transmission antenna and wave sensor. The main hull is surrounded by four 3D printed PETG secondary hulls filled with expanded polyurethane (Fig. 1). These secondary hulls give buoyancy stability to the entire assembly.

**Table 1:** Technical specifications of the EMAC buoy -V3.0-.

Dimensions (fully assembled)	4155 mm height, 615 mm diameter
Weight	48 Kg
Storage capacity	9 L
Standard battery bank	AGM 12 V 12Ah
GPS	Optional
Data transmission	3G / 4G / Satelital (Globalstar SmartOne C)
**Data logger**	
Memory capacity	3 months (optional external SD)
Numbers of A/D channels	8 (12 bits) (expandable to 16)
Number of digital channels	2
Power input	9 to 24 V (max. 2 Ah)
**Meteorological sensors**	
Anemometer	0 to 50 m s^−1^
Wind vane	0 to 360°
**Oceanographic sensors**	
Water temperature	−20 to 60 °C
Current velocity / Direction	0 to 5 m s^−1^ / 0 to 360°
Wave height / period / direction (inertial)	0 to 10 m / 0 to 50 s / 0 to 360°
**Working conditions**	
Air temperature	−20 to 60 °C
Wind velocity tolerance	40 m s^−1^
Wave height tolerance	8 m
Water flow tolerance	2 m s^−1^
Depth	100 m

3D printed ABS support pieces were carefully designed to fix mast, secondary hulls and different sensors (Fig. 1). These consist of four pieces similar to quarter-circumferences, which are assembled together forming a ring and fastened to the main hull with stainless steel bolts (Fig. 1). Each support is designed for a specific component, including the mast, secondary hulls and oceanographic measurement instruments. Specifically, the measurement instrument support has a set of special slots to accommodate the necessary instrument holders.

The aluminum mast of 2000 mm in height is attached to the main hull by a pair of 3D printed supports (Fig. 1). Safety light and meteorological sensors (wind velocity and direction) are mounted on a 3D printed structural support at the top of the mast (Fig. 1). Above the top end cap, there is a 12 V/20 W solar panel placed on an AISI 316 stainless steel plate (Fig. 1). This plate is supported on four threaded studs around the main hull (Fig. 1).

A counterweight (≅18 kg in total weight, 1000 mm in height) is mounted in the lower part of the main hull in order to help counter the drag forces for ensuring adequate measurements (Fig. 1). Both parts are joined by an AISI 316 stainless steel plate. The counterweight is composed of AISI 316 stainless steel.

### 2.2 Electronic parts: Data logger and sensors

**Fig. 2:**
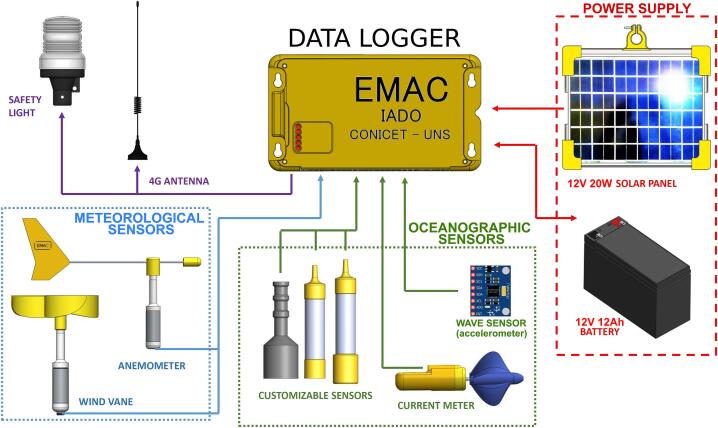
Interconnection diagram of the electronic parts.

Fig. 2 shows the interconnection diagram of the electronic part. The data logger is a compact and robust device which consists of a dual layer single board (Fig. 2). Its modular design allows multiple tasks such as processing, communication, data storage, power management, and includes a GPRS modem for connectivity. The case is 3D printed. This device, with nine inputs (eight of these are analogical) (Table 1), uses a 12-bit analog-to-digital microconverter to integrate existing commercial sensors into analogical outputs. The data logger has a RS232 connection port but can also use radio link to operate in remote regions. In addition, the data logger is equipped with a GSM/GPRS modem. The configuration software is user-friendly and it allows users to configure several parameters. For more details see Vitale et al. (2018) [10].

The buoy (basic configuration) is equipped with sensors for wave height, period and direction, current velocity and direction, water temperature as well as for meteorological variables (wind velocity and direction) (Fig. 2). The wave sensor combines a MPU6050 IMU/compass with a real time processing RB Pico board, while simultaneously making the data acquisition and processing. The water temperature sensor employs NTC thermistor. The electronics are housed in a 3D printed case filled with epoxy resin to make it highly waterproof. The current meter is based on a hall sensor that measures revolutions (i.e., 2 pulses per revolution). The rotor and the sensor body are ABS (or PETG) 3D printed pieces (Fig. 2). The wind velocity meter is based on a reflective optical sensor that measures revolutions (i.e., 4 pulses per revolution). The frequency-to-voltage conversion is managed by an integrated circuit technology (LM2907N). The rotor (Savonius-type) consists of an ABS (or PETG) 3D printed piece (Fig. 2); the sensor body is composed of stainless steel and Delrin. For more details about the mentioned sensors see Vitale et al. (2018) [10].

## 3 Design files summary

**Table 2:** Design file information (See Fig. 3).

**Component**	Item (*)	**Design file name**	**File type**	**Open-source license**	**Location of the file**
Central body					
Main hull	1	PVC_main_hull.STEP	STEP	GPL v3.0	https://data.mendeley.com/datasets/84fygz2txp
Top end cap	2	top_cap.STEP	STEP	GPL v3.0	https://data.mendeley.com/datasets/84fygz2txp
Bottom end cap	3	bottom_cap.STEP	STEP	GPL v3.0	https://data.mendeley.com/datasets/84fygz2txp
Top plate	4	top_plate.STEP	STEP	GPL v3.0	https://data.mendeley.com/datasets/84fygz2txp
Bottom plate	5	bottom_plate.STEP	STEP	GPL v3.0	https://data.mendeley.com/datasets/84fygz2txp
Stud − 1.5 m	6	−	−	−	
Waterproof connector (pair)	7	cable_marine_connector.STEP	STEP	GPL v3.0	https://data.mendeley.com/datasets/84fygz2txp
Inner cap	8	internal_tap.STEP	STEP	GPL v3.0	https://data.mendeley.com/datasets/84fygz2txp
Mast support section	9	mast_support_section.STEP	STEP	GPL v3.0	https://data.mendeley.com/datasets/84fygz2txp
Instrument support section	10	instrument_support_sect.STEP	STEP	GPL v3.0	https://data.mendeley.com/datasets/84fygz2txp
Counterweight	11	counterweight.STEP	STEP	GPL v3.0	https://data.mendeley.com/datasets/84fygz2txp
Solar panel subassembly					
12 V 20 W solar panel	12	sun_panel.STEP	STEP	GPL v3.0	https://data.mendeley.com/datasets/84fygz2txp
Solar panel support	13	panel_holder.STEP	STEP	GPL v3.0	https://data.mendeley.com/datasets/84fygz2txp
Mast holder in panel support	14	mast_panel_holder.STEP	STEP	GPL v3.0	https://data.mendeley.com/datasets/84fygz2txp
Panel retainer	15	panel_retainer.STEP	STEP	GPL v3.0	https://data.mendeley.com/datasets/84fygz2txp
Secondary hulls					
Secondary hull	16	hull.STEP	STEP	GPL v3.0	https://data.mendeley.com/datasets/84fygz2txp
Secondary hull cap	17	hull_cap.STEP	STEP	GPL v3.0	https://data.mendeley.com/datasets/84fygz2txp
Secondary hull support	18	Hull_support_sect.STEP	STEP	GPL v3.0	https://data.mendeley.com/datasets/84fygz2txp
Mast equipment					
Mast	19	alum_mast.STEP	STEP	GPL v3.0	https://data.mendeley.com/datasets/84fygz2txp
Mast holder	20	mast_holder.STEP	STEP	GPL v3.0	https://data.mendeley.com/datasets/84fygz2txp
Monitoring instruments support (central)	21	mast_equip_central.STEP	STEP	GPL v3.0	https://data.mendeley.com/datasets/84fygz2txp
Monitoring instruments support (side)	22	mast_equip_side.STEP	STEP	GPL v3.0	https://data.mendeley.com/datasets/84fygz2txp
Safety light cover	23	safety_ligth_cover.STEP	STEP	GPL v3.0	https://data.mendeley.com/datasets/84fygz2txp
Safety light holder	24	dafety_light_holder.STEP	STEP	GPL v3.0	https://data.mendeley.com/datasets/84fygz2txp
Anemometer subassembly	25a	anemometer_propeller.STEP	STEP	GPL v3.0	https://data.mendeley.com/datasets/84fygz2txp
	25b	shaft.STEP	STEP	GPL v3.0	https://data.mendeley.com/datasets/84fygz2txp
	25c	body_1.STEP	STEP	GPL v3.0	https://data.mendeley.com/datasets/84fygz2txp
	25d	body_2.STEP	STEP	GPL v3.0	https://data.mendeley.com/datasets/84fygz2txp
	25e	body_3.STEP	STEP	GPL v3.0	https://data.mendeley.com/datasets/84fygz2txp
	25f	inner_encoder.STEP	STEP	GPL v3.0	https://data.mendeley.com/datasets/84fygz2txp
	25 g	wire_plug.STEP	STEP	GPL v3.0	https://data.mendeley.com/datasets/84fygz2txp
Wind vane subassembly	26a	vane_1.STEP	STEP	GPL v3.0	https://data.mendeley.com/datasets/84fygz2txp
	26b	vane_2.STEP	STEP	GPL v3.0	https://data.mendeley.com/datasets/84fygz2txp
	26c	vane_3.STEP	STEP	GPL v3.0	https://data.mendeley.com/datasets/84fygz2txp
	26d	shaft.STEP	STEP	GPL v3.0	https://data.mendeley.com/datasets/84fygz2txp
	26e	body_1.STEP	STEP	GPL v3.0	https://data.mendeley.com/datasets/84fygz2txp
	26f	body_2.STEP	STEP	GPL v3.0	https://data.mendeley.com/datasets/84fygz2txp
	26 g	body_3.STEP	STEP	GPL v3.0	https://data.mendeley.com/datasets/84fygz2txp
	26 h	wire_plug.STEP	STEP	GPL v3.0	https://data.mendeley.com/datasets/84fygz2txp
	26j	vane_rod.STEP	STEP	GPL v3.0	https://data.mendeley.com/datasets/84fygz2txp
Inner electronics					
Datalogger subassembly	27a	dataloger_base.STEP	STEP	GPL v3.0	https://data.mendeley.com/datasets/84fygz2txp
	27b	datalogger_support.STEP	STEP	GPL v3.0	https://data.mendeley.com/datasets/84fygz2txp
	27c	datalogger_top.STEP	STEP	GPL v3.0	https://data.mendeley.com/datasets/84fygz2txp
	27d	EMAC2.0_PCB.STEP	STEP	GPL v3.0	https://data.mendeley.com/datasets/84fygz2txp
4G Antenna	28	4G_Antenna.STEP	STEP	GPL v3.0	https://data.mendeley.com/datasets/84fygz2txp
Current meter subassembly					
Current meter	29a	current_met_propeller.STEP	STEP	GPL v3.0	https://data.mendeley.com/datasets/84fygz2txp
	29b	current_met_cap.STEP	STEP	GPL v3.0	https://data.mendeley.com/datasets/84fygz2txp
	29c	current_met_body.STEP	STEP	GPL v3.0	https://data.mendeley.com/datasets/84fygz2txp
	29d	current_met_shaft.STEP	STEP	GPL v3.0	https://data.mendeley.com/datasets/84fygz2txp
	29e	current_met_tip.STEP	STEP	GPL v3.0	https://data.mendeley.com/datasets/84fygz2txp
	29f	current_met_holder.STEP	STEP	GPL v3.0	https://data.mendeley.com/datasets/84fygz2txp
Other specifications					
Instrument holder	30	inst_holder.STEP	STEP	GPL v3.0	https://data.mendeley.com/datasets/84fygz2txp
Buoy (complete assembly)	−	complete_buoy.STEP	STEP	GPL v3.0	https://data.mendeley.com/datasets/84fygz2txp
EMAC PCB	−	https://emac2.0_pcb_march_2025.zip/	−	GPL v3.0	https://data.mendeley.com/datasets/84fygz2txp
EMAC Firmware	−	https://firmware_v1.3.zip/	−	GPL v3.0	https://data.mendeley.com/datasets/84fygz2txp
Control Software (files)	−	Mercury 1.27.zip	−	GPL v3.0	https://data.mendeley.com/datasets/84fygz2txp
Control Software Manual	−	MercuryManual.pdf	−	GPL v3.0	https://data.mendeley.com/datasets/84fygz2txp

(*) For subitems 25a, 25b, 25c…etc. please see Fig. 4, Fig. 5, Fig. 6, Fig. 7.

## 4 Bill of materials summary

**Table 3:** List of hardware to be purchased (See Fig. 3).

**Component**	Item (*)	**Quantity**	**Material type**	**Unitary Cost (unit or meter)**	**Total cost**
Central body					
Main hull	1	1	PVC Pipe 200 mm	165	165
Top end cap	2	1	ABS 3D Printed (100 % infill)	7.9	7.9
Bottom end cap	3	1	ABS 3D Printed (100 % infill)	6.7	6.7
Top plate	4	1	AISI 316 steel (thickness 2 mm, laser cutting)	37.5	37.5
Bottom plate	5	1	AISI 316 steel (thickness 2 mm, laser cutting)	37.5	37.5
Stud − 1.5 m	6	4	AISI 316 steel (hexagonal)	51	204
Waterproof connector (pair)	7	6	PVC	67.5	405
Inner cap	8	1	ABS 3D Printed (100 % infill)	0.42	0.42
Mast support section	9	2	ABS 3D Printed (70 % infill)	3.4	3.4
Instrument support section	10	10	ABS 3D Printed (70 % infill)	3	30
Counterweight	11	1	AISI 316 steel (laser cutting)	750	750
Solar panel subassembly					
12 V 20 W solar panel	12	1	−	82.5	82.5
Solar panel support	13	1	AISI 316 steel (thickness 2 mm, laser cutting)	67.5	67.5
Mast holder in panel support	14	1	ABS 3D Printed (70 % infill)	0.8	0.8
Panel retainer	15	4	ABS 3D Printed (70 % infill)	1.3	5.2
Secondary hulls					
Secondary hull	16	4	PETG 3D Printed (100 % infill)	20.7	82.8
Secondary hull cap	17	4	ABS 3D Printed (40 % infill)	0.5	2
Secondary hull support	18	8	ABS 3D Printed (100 % infill)	2.5	20
Mast equipment					
Mast	19	1	Aluminium	30	30
Mast holder	20	2	ABS 3D Printed (70 % infill)	0.9	1.8
Monitoring instruments support (central)	21	1	ABS 3D Printed (70 % infill)	1.8	1.8
Monitoring instruments support (side)	22	2	ABS 3D Printed (70 % infill)	1.3	2.6
Safety light cover	23	1	Polycarbonate	18	18
Safety light holder	24	1	ABS 3D Printed (70 % infill)	1.3	1.3
Anemometer subassembly	25a	1	ABS 3D Printed (70 % infill)	211.5	211.5
	25b	1	Stainless steel rod (4 mm)		
	25c	1	PTFE		
	25d	1	AISI 316		
	25e	1	PTFE		
	25f	1	ABS 3D Printed (70 % infill)		
	25 g	1	−		
Wind vane subassembly	26a	1	ABS 3D Printed (70 % infill)	285	285
	26b	1	ABS 3D Printed (70 % infill)		
	26c	1	ABS 3D Printed (70 % infill)		
	26d	1	Stainless steel rod (4 mm)		
	26e	1	PTFE		
	26f	1	AISI 316 steel		
	26 g	1	PTFE		
	26 h	1	−		
	26j	1	Stainless steel rod (4 mm)		
Inner electronics					
Datalogger subassembly	27a	1	ABS 3D Printed (70 % infill)	345	345
	27b	1	ABS 3D Printed (70 % infill)		
	27c	1	ABS 3D Printed (70 % infill)		
	27d	1	−		
4G Antenna	28	1	−	15	15
Current meter subassembly					
Current meter	29a	1	ABS 3D Printed (100 % infill)	180	180
	29b	1	ABS 3D Printed (100 % infill)		
	29c	1	ABS 3D Printed (100 % infill)		
	29d	1	Stainless steel rod (4 mm)		
	29e	1	ABS 3D Printed (100 % infill)		
	29f	1	ABS 3D Printed (70 % infill)		
Other parts					
Instrument holder	30	4	ABS 3D Printed (70 % infill)	0.4	1.6
Wave sensor (accelerometer)	−	1	ABS 3D Printed parts (70 and 100 % infill); stainless steel rod (4 mm); others	135	135
Water temperature sensor	−	1	ABS 3D Printed parts (70 and 100 % infill); stainless steel rod (4 mm); others	165	165
12 V 12Ah Battery	−	1		90	90
Cable	−	16 m		3	48
Paint	−	1 (320 g)	−	10.5	10.5
Expanded Polyurethane	−	2 (2700 g)	−	22.5	45
Truss head machine screws, washers & nuts (5 mm x 40 mm)	−	12	AISI 304 steel	5	60
Truss head machine screws, washers & nuts (5 mm x 50 mm)	−	48	AISI 304 steel	6	288
Truss head machine screws, washers & nuts (5 mm x 80 mm)	−	2	AISI 304 steel	7	14
Hexagonal bolts, washers & nuts (6 mm x 25 mm)	−	4	AISI 316 steel	6	24
nuts (10 mm nominal size)	−	40	AISI 316 steel	0.6	24
washers (10 mm nominal size)	−	40	AISI 304 steel	0.9	36
Other supplies	−	−			450
				Total Cost	4391.3

(*) For subitems 25a, 25b, 25c…etc. please see Fig. 4, Fig. 5, Fig. 6, Fig. 7.

**Fig. 3:**
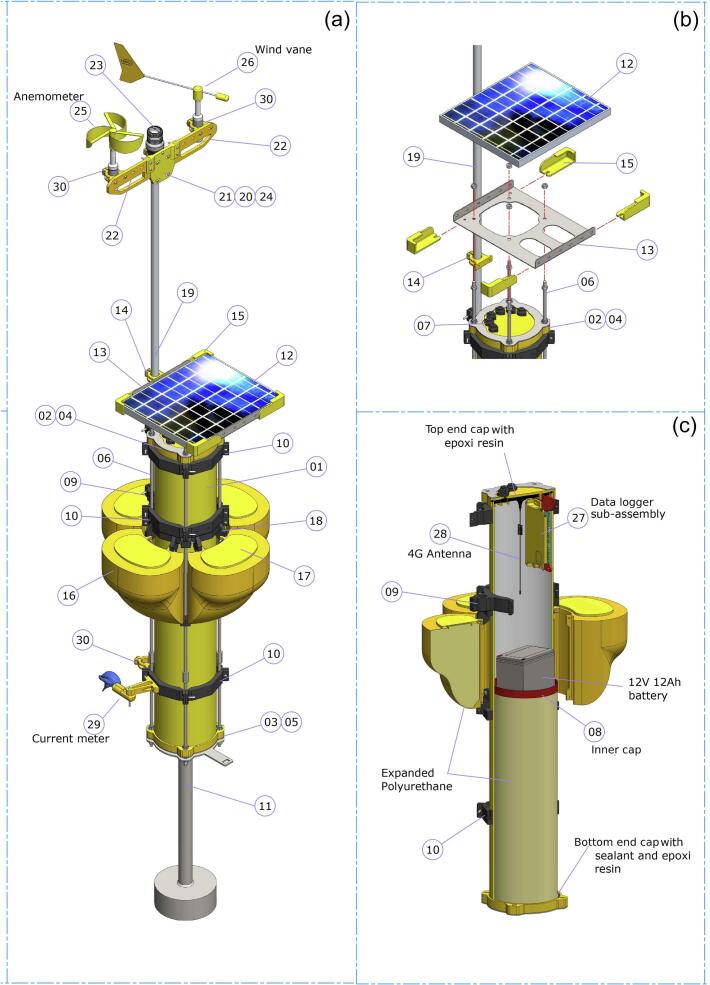
Constitutive parts of the EMAC buoy -V3.0-. General view of the buoy (a); view of the solar panel and its support (b); and view of the inner part of the main hull (c). See Table 2.

## 5 Build instructions

Descriptions of the build instruction steps will be detailed below. In general, parts assembly will be explained in order from smallest to largest. For the instructions belonging to commercial parts, users must implement manufacturers' specifications (Table 2 and Table 3, Fig. 3).

### 5.1 3DP of the parts

Adapted commercial 3D printers available at the Instrumental Laboratory of the IADO were used to create the above-mentioned 3D printed parts. At printing time, special environment conditions are required to prevent plastic-related issues such as warping or cracking due to thermal gradients. Therefore, enclosures (40 cm height cm x 35 cm width x 75 cm length) composed of polystyrene and acrylic were put around the printers. The 3DP configurations in terms of infill density percentage and pattern, layer thickness and printing speed were subject to trial and error for assessing the capacity to withstand loads and the surrounding space behavior. Most parts were printed at 70 % infill density using a tri-hexagonal pattern and a layer thickness of 0.2 mm ensuring adequate adhesion and, therefore, resulting in strong parts with low time-consumption. There are specific parts that require different configurations; for example, the end caps must be printed at 100 % infill density; the propeller of the current meter requires a layer thickness of 0.15 mm or less to achieve a smooth surface. Concerning the printing speed, it is recommended speeds below 80 mm min^−1^. Finally, the choice of ABS or PETG material was based on trial and error procedures during the design stage, in order to achieve the best cost-effectiveness and optimization. Characteristics that support the 3DP are summarized in Table 3.

### 5.2 Top end cap and data logger subassembly

1. Place the EMAC PCB (Fig. 4 -subitem 27d-) and the power switch into the 3D printed data logger case (Fig. 4 -item 27a-); close the case using M4 screws. Finally, attach the data logger case to the datalogger support (Fig. 4 -subitem 27b-) using M4 screws.

**Fig. 4:**
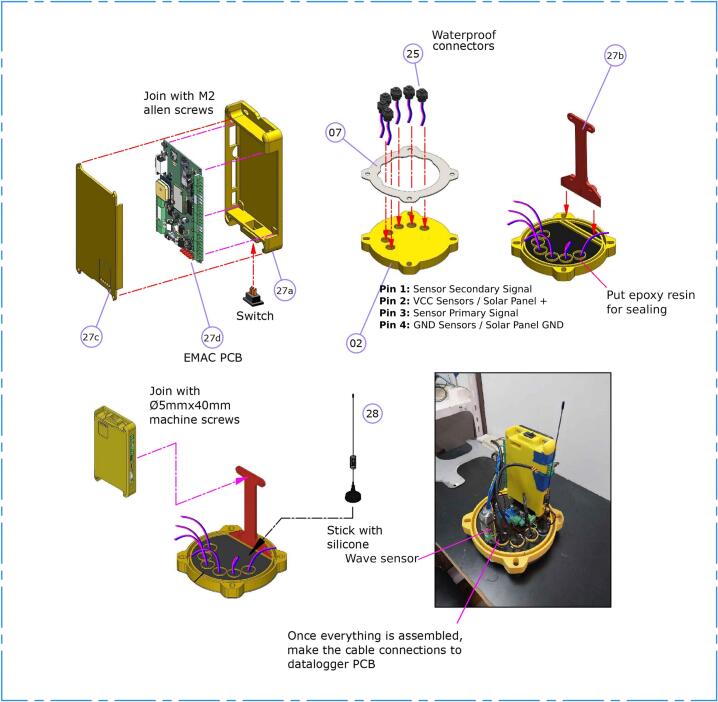
Top end cap and data logger subassembly.
2. Solder the cables onto the waterproof connectors (Fig. 4 -item 25-); then, place the waterproof connectors into the top end cap (Fig. 4 -item 25-) applying cyanoacrylate (Fig. 4, upper middle).
3. Apply epoxy resin in the inner face of the top end cap to provide a watertight sealing. Then, glue the stainless steel plate to the end cap using silicon. Insert an O-ring sealing into the end cap (Fig. 4, upper right).
4. Mount the data logger, antenna (Fig. 4 -item 28-) and wave sensor in the top end cap (Fig. 4, lower left).
5. Connect the waterproof connectors to the data logger to link the external equipment (Fig. 4, lower right).

### 5.3 Mast and its equipment subassembly

1. The anemometer (Fig. 5 -item 25-) and wind vane (Fig. 5 -item 26-) require manufactured parts by using machine turning and 3DP techniques. All components are available in the repository. The anemometer uses a slotted wheel with an encoder along with an optical sensor. The signals are collected by the data logger and processed to determine the wind speed. Calibrations were made in the Fluid Mechanics Laboratory of the National University of the South (Universidad Nacional del Sur -UNS-). The wind vane uses a potentiometer (model number 6639S-1-103). Both sensors use 604ZZ bearings. For more details see Vitale et al. (2018) [10].

**Fig. 5:**
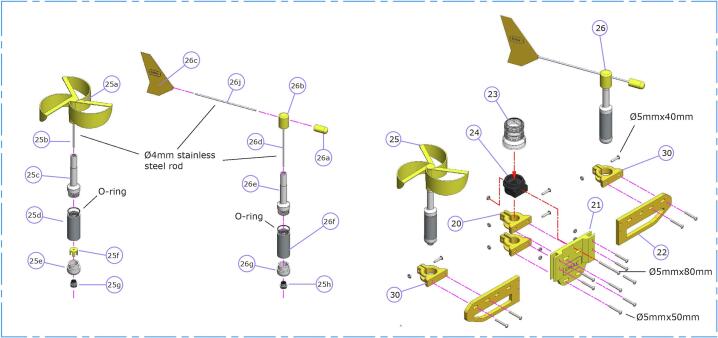
Mast and its equipment subassembly.
1. The 3D printed pieces (Fig. 5, right -item 22-) must be bolted to the central piece (Fig. 5, right -item 21-) using M5×50 stainless steel bolts. The instrument holder (Fig. 5 -item 30-) and mast holder (Fig. 5 -item 20-) should then be mounted using M4 screws. Adjustments to the instruments or mast should later be made with M5×40 stainless steel bolts (Fig. 5, right).
2. The safety light cover (Fig. 5 -item 23–) is contained in a support (Fig. 5 -item 24-) that must be attached to the central piece (Fig. 5 -item 21-) with a M4×80 stainless steel screw.

### 5.4 Secondary hulls subassembly

1. Each secondary hull (Fig. 6, left) must be filled with expanded polyurethane (8 L -fully expanded-). Excess polyurethane foam must be cut off, and the end cap (Fig. 6 -item 17-) should be glued using silicone, ensuring its waterproofing.

**Fig. 6:**
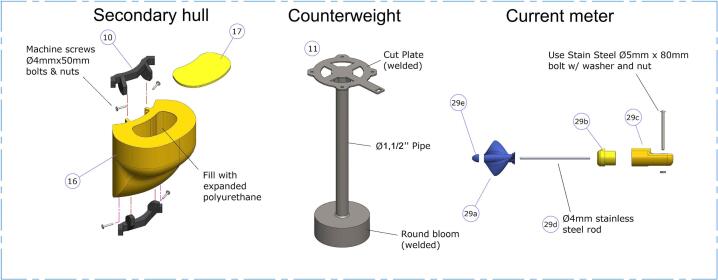
Secondary hull, counterweight and current meter assembly.
2. Each secondary hull must be bolted to two hull supports (Fig. 6 -item 10-) using M4×50 machine screws with nuts.

### 5.5 The counterweight

The counterweight (Fig. 6, middle -item11-) serves the function of stabilizing the buoy and of balancing the tipping moment by lowering its center of gravity. It consists of a laser cut stainless steel plate, ND 1.5′’ pipe and a 15-kilogram counterweight. All components must be welded.

### 5.6 The current meter

1. The current meter consists of 3D printed and commercially available components (Fig. 6, right). Calibrations were made in the Fluid Mechanics Laboratory of the UNS.
2. The head (Fig. 6 -subitem 29b-) houses a Hall effect sensor positioned in the front wall and embedded in epoxy resin to protect it from saltwater.
3. The body (Fig. 6 -subitem 29c-) contains the cable connected to the sensor, passing through it. The head (Fig. 6 -subitem 29b-) must be fitted and bonded to the body.
4. A 4 mm-diameter rod must be inserted and bonded at the center of the assembly. The propeller (Fig. 6 -subitem 29a-) rotates freely in the rod, while the tip (Fig. 6 -subitem 29e-) must be fixed to prevent axial displacement of the propeller.
5. The propeller has two holes for inserting 4 mm diameter neodymium magnets, which activate the Hall effect sensor (a3144) as they rotate. The pulses are registered by the data logger, where they are interpreted as current speed.

### 5.7 Main hull assembly

1. The bottom end cap (Fig. 7a -item 03-) must be bonded to the bottom plate (Fig. 7a -item 05-) using silicone. The epoxy resin should also be applied at the center of the end cap to ensure waterproofing. In the peripheral groove, where the 200 mm PVC pipe fits, resin should be applied for bonding, but the pipe must be inserted while the resin is still fresh. This will not only provide waterproofing but also bond the pipe to the end cap (Fig. 7a, left).

**Fig. 7:**
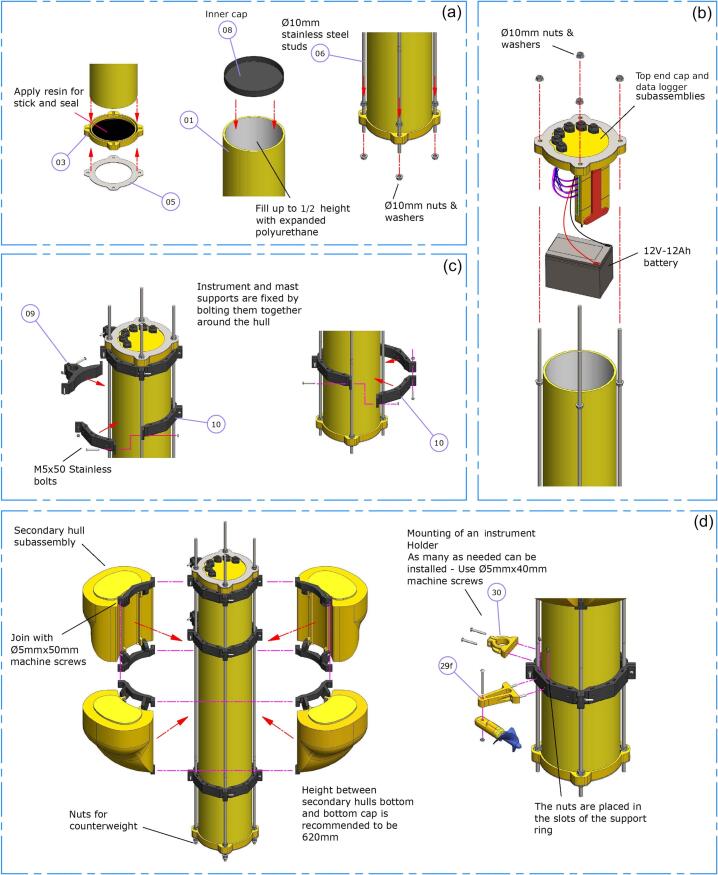
Main hull assembly.
2. Once the previous step is completed, expanded polyurethane should be poured inside the pipe (once dry, it should fill half of the pipe). Before the polyurethane dries, the inner cap (Fig. 7a -item 08-) must be placed and bonded in position to create the internal compartment of the main hull (Fig. 7a, middle).
3. The studs (Fig. 7a -item 06-) must be placed in the holes of the bottom end cap (Fig. 7a -item 03-) using a double-nut system and washers. A free thread length of approximately 40 mm should be left below the lower nut (Fig. 7a, right).
4. The battery must be placed inside the main hull, on the inner cap (Fig. 7a -item 08-), and it is connected to the data logger (Fig. 7b).
5. At the subassembly step of the top end cap and data logger with the pipe, it must be sealed using an O-ring (6 mm); then, tighten the four nuts onto each stud. To ensure parallelism of the surfaces, it is recommended to tighten bolts by following a criss-cross procedure (Fig. 7b).

### 5.8 Support rings and secondary hulls mounting

1. The assembly of the EMAC buoy -V3.0- consists of two mast supports (Fig. 7c -item 09-) and one instrument support ring (composed of four pieces) (Fig. 7c -item 10-). These must surround the PVC pipe and be fastened together using M4×50 machine screws, washers and nuts (Fig. 7c).
2. Secondary hull subassemblies are installed similarly to the other support rings. At a height of 620 mm from the base of the bottom end cap (i.e., base of the secondary hull), it is recommended to ensure proper buoyancy (Fig. 7d, left)

### 5.9 Current meter and instrument Mounting

1. The current meter must be mounted on its support (Fig. 7d -subitem 29f-) using a M4×80 stainless steel bolt and a double-nut system (Fig. 7d, right).
2. The attachment of the current meter holder (Fig. 7d -item 30-) as well as any instrument holder is done using M4×40 stainless steel bolts. The nuts must be placed in the slots of the supports while the bolts must be inserted from the outside, ensuring the fixation of the holders (Fig. 7d, right).

It is important to remark that the instrument support slots were designed for 3 mm nuts; this can be modified using the STEP file provided in the repository. Additional instrument holders can be easily mounted on the main hull; these holders can be modified using STEP file provided in the repository for different sensors to fit. It is also possible to add more support rings to install additional equipment.

### 5.10 Final assembly

1. Place the mast (Fig. 3a -item 19-) in the mast supports (Fig. 3a -item 09-).
2. Place the solar panel support (Fig. 3b -item 13-) using a double-nut system, approximately 180 mm from the top end cap (Fig. 3b -item 02-), ensuring parallel alignment.
3. The panel-mast holder (Fig. 3b -item 14-) must be mounted using two M5×50 machine screws and nuts (Fig. 3b).
4. Install the solar panel (Fig. 3b -item 12-) and the retainers (Fig. 3b -item 15-) using M6×25 hex bolts and nuts positioned in the panel slots.
5. Once everything is assembled, connect the instruments, mount the meteorological equipment at the top of the mast (Fig. 3b -item 19-), and then attach the counterweight.
6. Power on the buoy. For this, the solar panel connector must be connected to its respective waterproof conector (bridge between the pin 1 and 3, Fig. 4, upper -item 25-).

## 6 Operation instructions

**Fig. 8:**
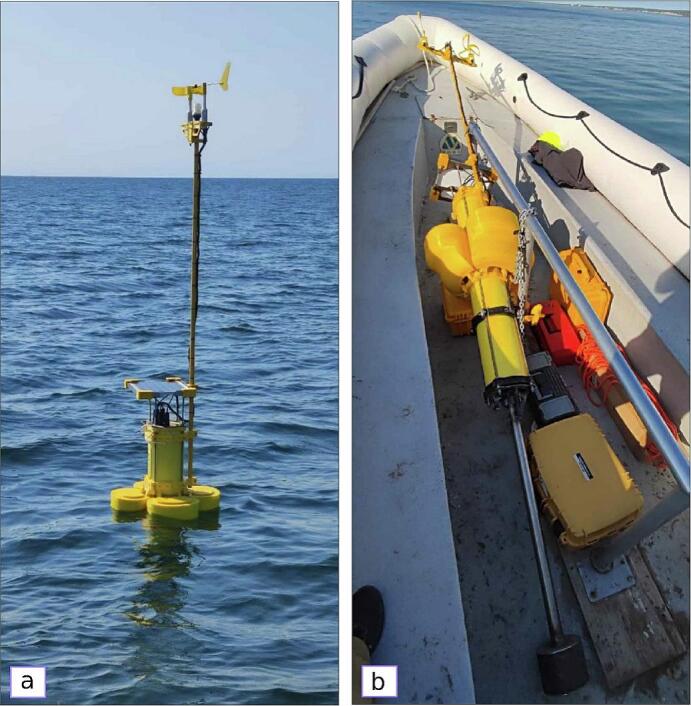
Deployment (a) and transfer by boat (b) of the EMAC buoy -V3.0-.

The deployment of the EMAC buoy -V3.0- is similar to that of commercial buoys (Fig. 8a,9). Based on experiences from previous versions, the following suggestions are recommended:

1. Because of the small size of the buoy, it can be easily transferred by land and sea, either in the vertical or horizontal position. A small-sized transfer boat is suitable for moving and deploying the buoy (Fig. 8b).
2. Prepare an anchor preferably built in concrete (≅300 kg weight).
3. Attach an AISI 316 stainless steel chain (10 mm thickness) to the anchor using bow shackles. The chain is tied to a 16 to 20mm diameter rope connected to a marking buoy to indicate the location for further support (Fig. 9).

**Fig. 9:**
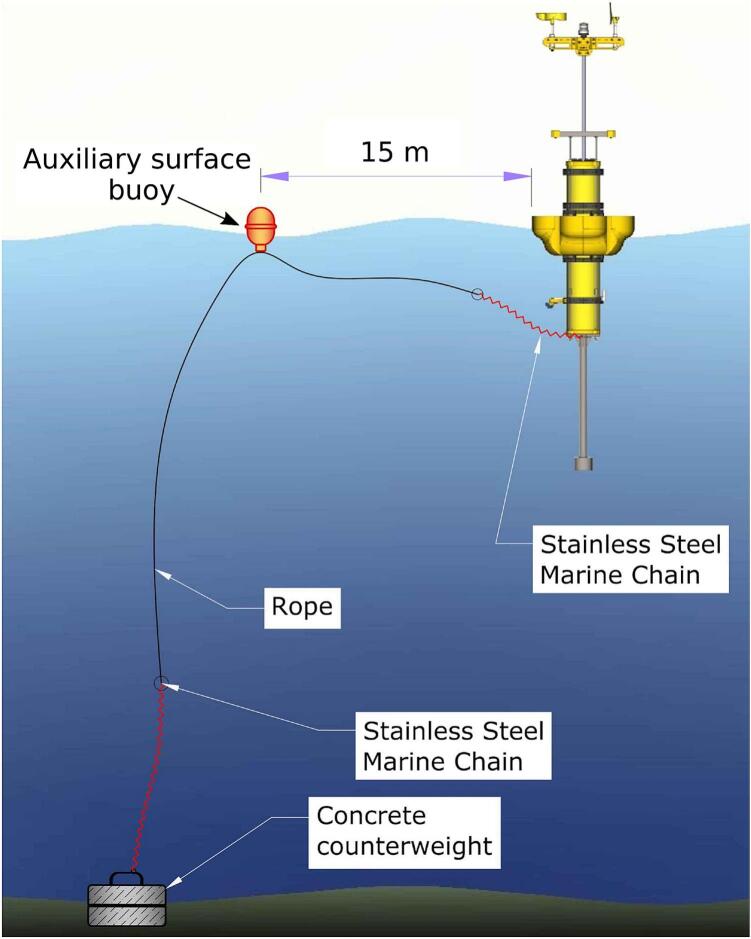
Deployment scheme of the EMAC buoy -V3.0-.
4. Once the buoy and the marking buoy are deployed in the water, connect them by means of a chain (similar to the former); the length of the chain is about three times the depth (Fig. 9).
5. The maintenance tasks are mainly performed on the sensors (biofouling, see Vitale et al. (2018) [10]) and, eventually, on the solar panel (cleaning of the surface) and battery (replacement). The maintenance tasks can be easily solved in situ with a small boat employing up to two people.

## 7 Validation and characterization

**Fig. 10:**
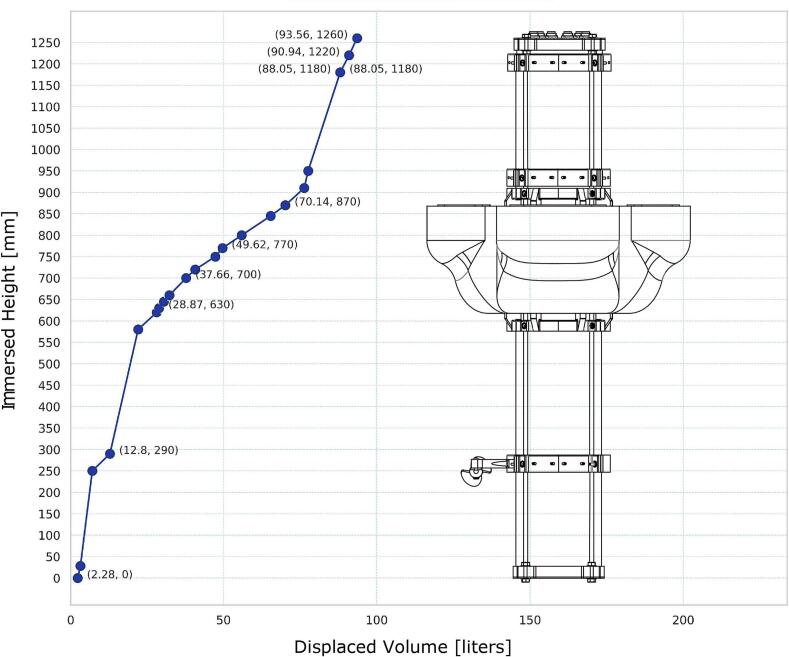
Displaced water volume of the EMAC buoy -V3.0- at different immersed heights.

The dynamic behavior of the buoy system implies an important challenge in its design. A numerical approximation of the displaced water volume was made using FreeCAD Graphic User Interface [18] (Fig. 10); the additional equipment and sensors (except current meter) were not considered in the calculation. The graph shows a gradual displacement of water volume in the vertical direction. Fully equipped, the buoy has between 15 and 20 L of remaining operational buoyancy. Also, if needed, the round bloom (counterweight) can be replaced by an ADCP, and the buoy balance will be the same.

As above mentioned, the Emac buoy -V3.0- considered in this study is moored in the northwestern part of the San Matías Gulf, on the northern Patagonian Continental Shelf, Argentina (40°53′28.0″S 65°03′57.1″W). In order to give an assessment of the performance of the buoy, a comparison between the observed data from the buoy and the modeled data was carried out; modeled data were used since in-situ observed data is not available in the study region. Database of the WAVERYS model (GLOBAL_REANALYSIS_WAV_001_032, see details in E.U. Copernicus Marine Service Information [19]) was used regarding the wave height parameter; the node of the model (40°55′0″S 65°0′0″W) is located 6.1 km southeast of the buoy. In addition, the ERA5 global reanalysis [20] was used regarding the wind velocity parameter; the node of the model (40°50′2.40″S 64°55′1.20″W) is located 14 km northeast of the buoy. Three statistical indexes (root mean square error -RMSE-, correlation coefficient -R- and determination coefficient -R^2^-) were applied for these assessments.

**Fig. 11:**
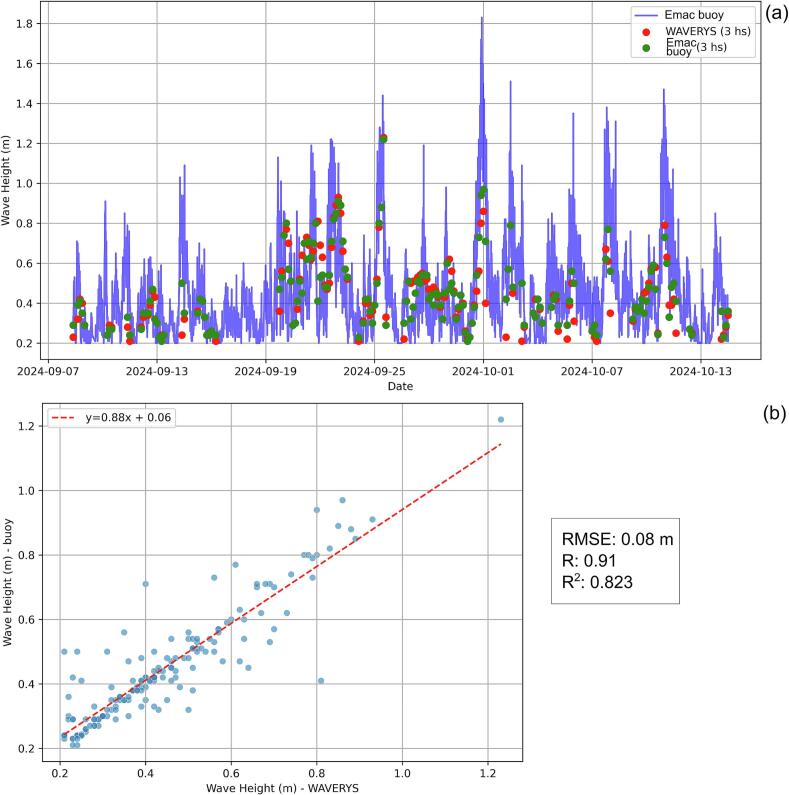
Observed and modeled time series of H_S_ (a), and R^2^ of the observed vs. modeled time series of H_S_ (b).

**Fig. 12:**
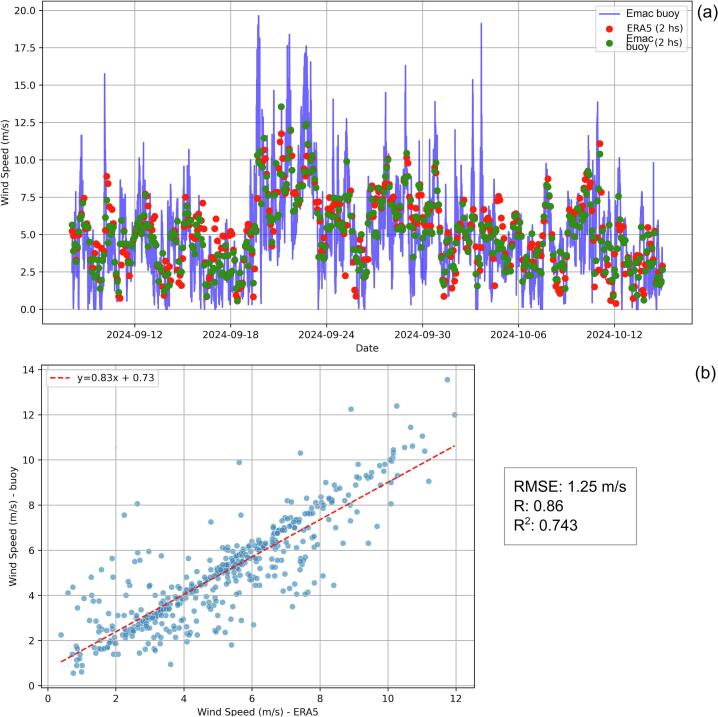
Observed and modeled time series of wind velocity (a), and R^2^ of the observed vs. modeled time series of wind velocity (b).

As can be seen in Fig. 11, the observed and modeled time series of H_S_ showed a good general agreement during the analyzed period. The statistical index showed a RMSE of 0.08 m, a R of 0.91 and a R^2^ of 0.82 (Fig. 11b). Only during the strongest storm of 1 October 2024 in which the H_S_ reached more than 1.8 m (buoy data), the agreement was slightly weaker (Fig. 11a). Here, it is convenient to mention that according to Salimbeni et al. (2024) [21], the WAVERYS model is able to satisfactorily capture the temporal variability, although, the highest H_S_ are slightly underestimated during extreme conditions. With respect to the wind velocity, a good agreement between the observed and modeled time series was found (Fig. 12). The RMSE was 1.25 m/s; the coefficients of correlation and determination were 0.86 and 0.74, respectively (Fig. 12b).

**Fig. 13:**
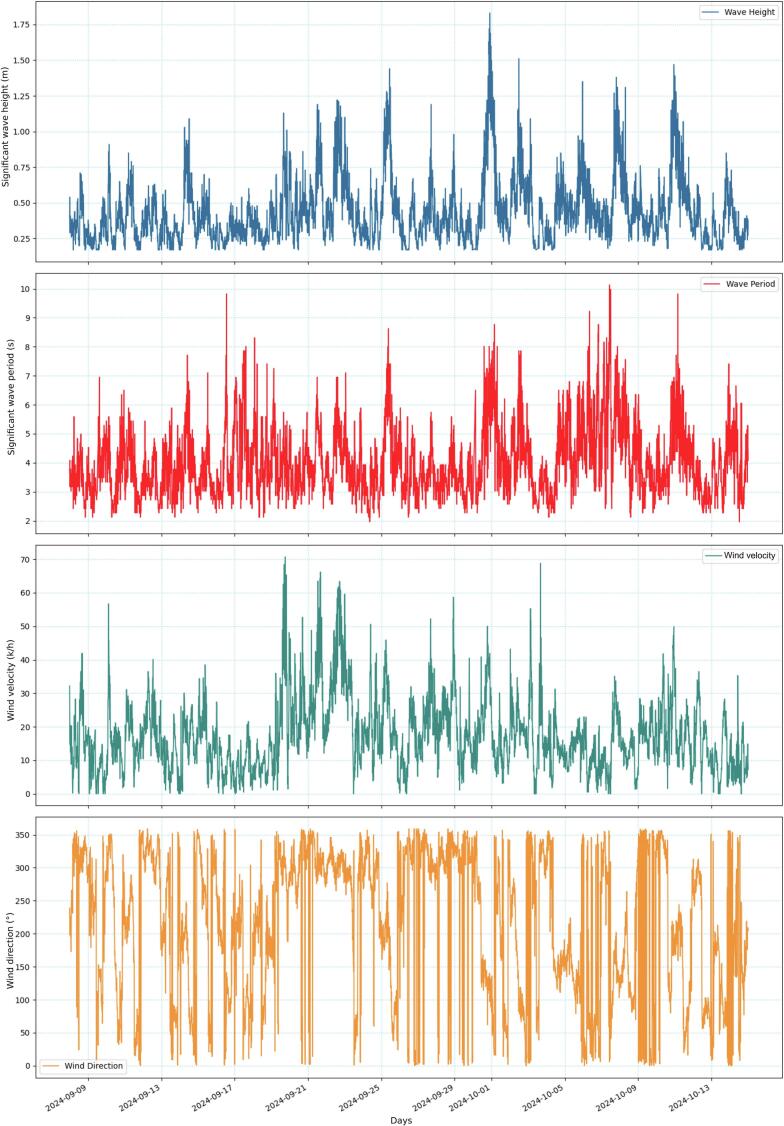
Observed wave (H_S_ and T_S_) and wind (velocity and direction) time series.

**Fig. 14:**
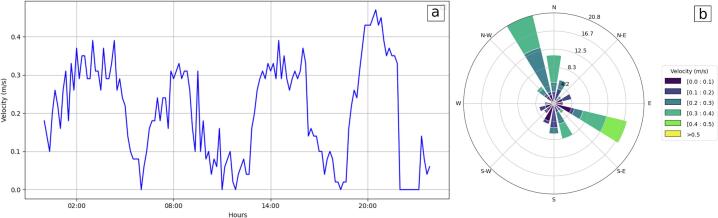
Observed surface current velocity (a) and direction (b) during the storm event (10/01/2024).

Meteorological (i.e., wind velocity and direction) and oceanographic (i.e., wave height and period) time series from the buoy were jointly analyzed to determine its performance (Fig. 13). Here, the storm event of 1 October 2024, in which the significant wave heights (H_S_) and periods (T_S_) are larger, was particularly considered. All the meteorological (wind velocity) and oceanographic (H_S_, T_S_) variables responded, as expected, to the strongest events (Fig. 13). For instance, the variables described a typical storm pattern with wind velocities reaching more than 50 km h^−1^ and directions from the south, and with H_S_ and T_S_ reaching up to 1.8 m and 9 s, respectively. Therefore, the assembled sensors in the buoy taken together and adequately calibrated provided a good depiction of the sea-atmosphere system for a given time period. Fig. 14 presents the observed surface current velocity and direction during the storm event. The velocity curve shows a pattern clearly related to the local tidal cycle (semidiurnal) and is visibly influenced by the meteorological conditions (Fig. 14a). The maximum velocity reached 0.5 m s^−1^, moving from the SE sector (Fig. 14b).

## 8 Conclusions

In this study, the EMAC buoy -V3.0- design for coastal monitoring was presented. It is characterized by a low-cost, small and compact platform, which was built almost entirely on 3DP technology in a modular way, providing the advantages of simplicity, flexibility and robustness. In particular, it should be noted that the engineering design and development of 3D pieces for support and attachment (e.g., holders, rings) provides good applicability and performance.

The cost of the proposed buoy (USD 4392) includes the material, purchased commercial parts as well as the electronic manufacturing, lathe machining, welding and metal cutting as labor costs. It must be mentioned that the assembling and 3D printing tasks were not considered in the cost calculation. This cost is much lower than commercially available buoys and, therefore, becomes suitable for scientific purposes, even more for developing countries.

In order to give an approach to the performance of the buoy, a comparison between the observed buoy data and the modeled data (WAVERYS -wave- and ERA5 -wind-) was carried out. As a result, the observed versus modeled time series of H_S_ and wind velocity showed a good general agreement. Likewise, the assembled sensors in the buoy taken together and adequately calibrated can provide a good depiction of the sea-atmosphere system. It should be mentioned that the EMAC buoy -V3.0- can be deployed in other water bodies as long as the tolerance conditions are satisfied.

## CRediT authorship contribution statement

**Simón F. Nogueira:** Writing – review & editing, Visualization, Supervision, Methodology, Investigation, Formal analysis, Conceptualization. **Alejandro J. Vitale:** Writing – review & editing, Visualization, Validation, Supervision, Software, Methodology, Investigation, Funding acquisition, Formal analysis, Conceptualization. **Sibila A. Genchi:** Writing – review & editing, Writing – original draft, Validation, Investigation. **Agustina Roth:** Visualization, Validation, Data curation. **Steven Martínez Vargas:** Software. **Agustin Siben:** Software. **Lucas Nuciari:** Methodology. **Gerardo M.E. Perillo:** Writing – review & editing, Supervision, Investigation, Funding acquisition.

## Declaration of competing interest

The authors declare that they have no known competing financial interests or personal relationships that could have appeared to influence the work reported in this paper.
